# Automatic Translation and Enforcement of Cybersecurity Policies Using A High-Level Definition Language

**DOI:** 10.3390/e21121180

**Published:** 2019-11-30

**Authors:** Diego Rivera, Fernando Monje, Victor A. Villagrá, Mario Vega-Barbas, Xavier Larriva-Novo, Julio Berrocal

**Affiliations:** ETSI de Telecomunicación, Departamento de Ingeniería de Servicios Telemáticos, Universidad Politécnica de Madrid (UPM), Avda. Complutense 30, 28040 Madrid, Spain; f.monjer@alumnos.upm.es (F.M.); victor.villagra@upm.es (V.A.V.); mario.vega@upm.es (M.V.-B.); julio.berrocal@upm.es (J.B.)

**Keywords:** cybersecurity, security policies, automatic response systems, firewalls, risk assessment

## Abstract

The increasing number of cyber-attacks, their potential destructive capabilities, and the enormous threat they pose for organizations, require the constant design and development of new, faster, and easier to use systems to address them. The automation of security enforcement systems is one of the most important techniques for enabling a fast response to security challenges, but the complexity of security management might hinder the successful achievement of the desired security. Our proposal integrates the automatic enforcement of security rules based on intrusion detection systems with the definition of a high-level user-centered language for the definition of policies. We have designed a translation process from this language to specific network-wise and device-aware rules that can be installed and enforced. The deployment of these rules is determined by an automatic risk assessment process ruled by the detection system monitoring the network. This way, both the automation and easiness of use goals can be achieved using an integrated system. The solution was tested and validated in two different virtualized networks.

## 1. Introduction

The security requirements in organizations’ networks are becoming more and more demanding, and the complexity has increased accordingly in the last years. These requirements are derived from the emergence of new security threats that should be addressed as fast as possible and through processes as automated as possible. A good example of how complex it is to manage cybersecurity in certain scenarios is the generation, management, and enforcement of security policies for networks. There are many proposals and research focused on the generation of reliable, easy-to-use, and fast policy management and enforcement systems, but, despite the efforts, most of these proposals present important drawbacks in terms of complexity, thus making the design, management, and usage of policies a very difficult task. The complexity of this task might have consequences in the effectiveness of the network security measures and will definitely have a negative impact on the human and computer resources needed to maintain a secure network. Moreover, in many cases, the proposals might only focus on the policy generation mechanisms but do not determine the connection between the policy generation process and actual devices installed in the network that give information and alerts for possible security threats.

The definition of policies is usually a task which requires a high knowledge and skills from users. It requires the gathering of access control and other security requirements, the assessment of threats and associated risks, the actual generation of policies, and its deployment and enforcement through firewalls or other mechanisms that provide security functionalities. On the other hand, policy definition tasks can make use of the information retrieved by intrusion detection systems (IDS) and other automated processes, and integration with firewalls allow for a certain degree of automation in the policy generation.

Although there have been many proposals regarding the use of high-level languages in security policy enforcement, most of them are not intended to be used for real-time dynamic policy deployment, which is currently an important feature of security enforcement systems. Nevertheless, the emergence of automatic intrusion response systems (AIRS), jointly with IDSs, have propitiated the definition of more automated dynamic responses to the detected threats in current networks.

Our proposal aims to integrate the automatic security policy enforcement with the creation of a specific high-level human-readable language that can be automatically translated into hardware-specific security rules to be deployed in network devices depending on risk assessment. The usage of a technology-agnostic topology-unaware language eases the enforcement task. The integration of this translation system with automatic risk assessment, intrusion detection, and rule deployment favors an easier, less error-prone network security management.

The rest of this paper is organized as follows: In Section 2 we discuss the background technologies and related works related to the policy definition languages in cybersecurity environments. In Section 3 we define further our proposal and its development, including a description of each high-level document generated, the translation process and the integration with the IDS to create a complete AIRS. In Section 4 we apply the solution to different networking scenarios that demonstrate the validity and the advantages of the system. Finally, in Section 5 we discuss and explain our conclusions regarding the proposal and its application.

## 2. Related Work

There have been many efforts and proposals in recent years to provide high-level human-readable languages for security policy definition, and some of them are widely used in organizations’ management systems. The rationale behind the use of a high-level language to transform the security requirements in policies relies on the necessity of counting on a flexible yet easy-to-use method to explain and define each possible configuration of a wide range of heterogeneous network elements. This allows cybersecurity managers to count on a unified method for the definition of all the requirements in a given scenario. These languages are usually based on specific access control models, which determine the focus of the control system. Some of the most used models are RBAC (role-based access control) [1], ABAC (attribute-based access control) [2], CapBAC (capabilities-based access control), widely used in Internet of Things scenarios [3], CPBAC (conditional purpose-based access control) [4] or OrBAC (organization-based access control) [5]. Some of the most used high-level languages are based on RBAC, given that using roles to define the access to resources is a very common approach in organizations. On the other hand, in [6] for instance, the authors present a model based on OrBAC which aims to specify a policy description specification decoupled from the devices enforcing their application, but OrBAC models can be seen as a generalization of RBAC models with further abstraction levels.

The most widely used languages are described and classified in works such as [7], and more recently in [8]. In those works, the authors present the main features, advantages, and drawbacks of each language. From the languages referenced in those works, it is important to consider at least the following for their relevance and/or wide use:

- XACML (eXtensible Access Control Markup Language) [9]: It is, by far, the most used language in industry, and it is considered a de facto standard. It is based on the ABAC model and in XML (eXtensible Markup Language), although it provides support for RBAC, especially when using extensions of the language such as xfACL (eXtensible Functional Language for Access Control) [10].
- EPAL (Enterprise Privacy Authorization Language) [11]: Also based in XML and following a CPBAC model, it more focused on privacy than in any other task, and present a high degree of complexity in its implementation. IBM, the language’s designer company, has provided a specific toolkit to help mitigate the difficulties that arise when implementing it [12].
- VALID (Virtualization Assurance Language for Isolation and Deployment) [13]: It is a language defined to provide high-level security, aiming specifically to virtualized environments. This language is not based on XML and instead is based on a protocol called IF (Intermediate Format), which is part of the AVISPA project [14].
- Ponder [15]: Another declarative language based on the RBAC model, where the interaction between roles is defined as relationships. Although it is not XML-based, it can be translated into it. It defines various types of policies (for authorization, filtering, delegation, etc.).
- Rei [16]: Also a declarative language, it is focused on security and privacy enforcement. It might be seen as an RBAC-based language, but it extends the model allowing both individual and grouped roles. Its implementations must provide a dynamic authorization decision process.
- ASL (Authorization Specification Language) [17]: Another RBAC-based language, which defines authorization policies using a quadruple of users, roles, sets, and objects. Its decision-making process is rather simple and static and lacks flexibility.
- OSL (Obligation Specification Language) [18]: It is focused on resource usage control, allowing to determine the duration of permissions and other statements used in digital rights management (DRM).

An important aspect of these language’s implementations is that only some of them allow for a dynamic policy modification and enforcement process, which is a crucial part of modern security systems. A completely manual configuration of the policies might lead to incorrect configurations, mistakes and, above all, too-slow response time to the increasing threats that arise nowadays. To mitigate this problem, there have been proposals for adding automation in the detection and response to threats in a dynamic manner. These systems are usually called generically AIRS and rely on IDSs to gather information about possible intrusions and threats. These systems must be configured according to the specific context, requirements, and costs associated with the scenario where they are going to be deployed. For instance, the AIR proposed by Stakhanova et al. [19], is based on two main metrics: the comparison between the damage an intrusion could cause in the system and the damage that could be derived from the response, and the comparison between benefit and risk. On the other hand, Carver et al. [20] proposed AAIRS (adaptive, agent-based intrusion response system), based on the confidence level of the IDS used (that is, its number of false positives), the correct reactions to actual intrusions, and the success rate of past reactions. IDAM&IRS (intrusion detection alert management & intrusion response system) [21] is a more modern approach that associates preset responses to risk levels. Depending on the measured risk for a given intrusion and the risk related to the response, it acts accordingly. The use of ontologies to improve the behavior of the IRS [22]. A more detailed classification of AIRs and other intrusion response systems can be found in [23].

In general, the main drawback of AIRS systems is that they are not integrated with the policy definition languages and vice versa. This means that it is not always easy to define the required policies to be managed by the AIRS due to its complexity, making it necessary to manually configure the response system. Therefore, a proposal designed to allow the use of a high-level language to define policies that could be automatically enforced into an automated intrusion response system would integrate the advantages of both approaches.

Although SIEM (security information and event management) systems and IDS/AIR systems are technologies which not cover exactly the same aspects in the security field, their goals, functionalities, and processes are highly related. In fact, it is very common to use high-level languages for the definition of correlation rules in SIEM systems [24]. For instance in commercial SIEM solutions such as OSSEC (open source HIDS (Host-based Intrussion Detection Systems) SECurity) or OSSIM (open source security information management), an XML-based language is included for that task [25]. Other systems such as Prelude use Lua language [26], and in Esper, they use an event processing language (EPL) [27]. Nevertheless, In most SIEM systems, the languages are mainly focused on the construction of correlation rules which could aid in the detection of possible threats or intrusions, while the goal of our proposal is the definition of a language for the generation of security enforcement policies, related specifically with the access control through firewalls and other related network devices.

It is also important to consider in this section the relationship between these automatic security enforcement languages and software defined networking (SDN) technologies. SDN aims to improve network flexibility by separating the control and data planes and centralizing the network intelligence in a controller device. SDN languages such as OpenFlow can be seen as a high-level language for the definition or network routing rules, and therefore are highly related to the goal of our work. In fact, there is a wide research field regarding the relationship between SDN and security issues [28]. There are some proposals for the use of SDN-based rules to configure firewalls in SDN-enabled networks [29,30]. Moreover, SDN has been combined with IDSs and intrusion prevention systems (IPS) to improve security in networks [31]. Also, in a similar way to our proposal, SDN controllers have been used for security policy enforcement in specific scenarios [32] and for the detection of attacks such as DDoS (distributed denial of service) [33] and others [34] in that kind of network. There are also proposals for more generic intrusion detection and prevention systems for SDN-based networks [35], and the definition of security policies through these technologies has been also explored in works such as [36] or [37]. Although the use of SDN technologies is a very promising field in security, it requires the deployment of SDN-enabled devices in organizations’ networks and the modification of the current network management, which might have a high impact in terms of network reorganization and cost.

## 3. Proposed Solution

Our proposal aims specifically to combine both concepts and allow an easy policy definition and security configuration while automating the enforcement and response processes through an integrated AIRS. This would reduce the complexity from the user point of view and would contribute to the automation of the whole security process. Specifically, we propose a solution based on an RBAC model for the definition and configuration of policies through a high-level language. Then we have designed a mapping process to translate the high-level language into low-level rules that can be directly applied and enforced using firewalls and other network mechanisms. Our solution presents two main advantages from the reviewed literature proposals: First, as the high-level language is technology-agnostic, and the system core is also independent of the actual network devices, it is possible to integrate the solution in any organization’s network without a high cost in specific new technology installation. Also, in our proposal, each decision-making process is performed in each enforcement point, instead of using a centralized point like in other approaches (for instance, the XACML reference architecture described in [38]), which enables a decentralized process that increases its reliability.

**Figure 1:**
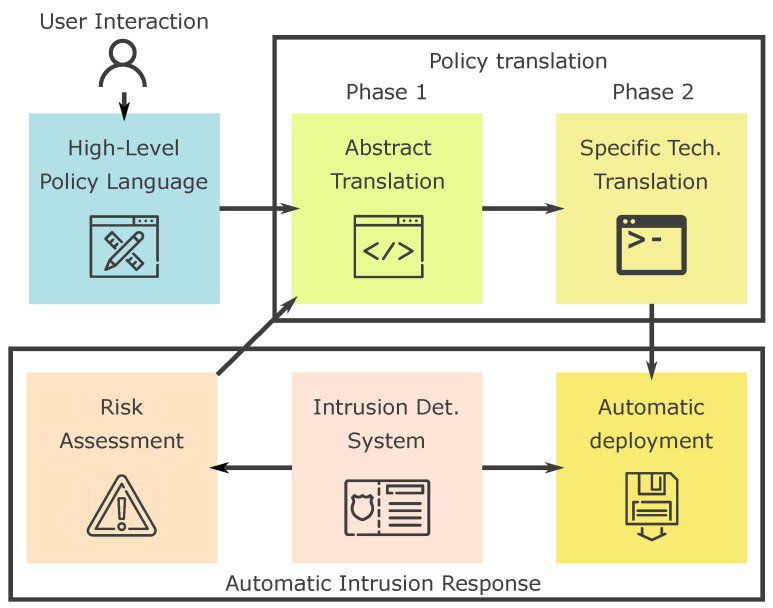
Main diagram of the proposed solution: The high-level language policies are translated using a two-phased mechanism into rules that can be automatically deployed. The intrusion detection system (IDS) automatically assesses the risk to decide which rules should be deployed and enforced.

The main components of our proposal are shown in Figure 1. The system is composed of a specifically designed human-readable high-level language that allow users to define policies through file editing or a graphical interface. Then, we have defined a two-phase translator to transform the high-level policies into an abstract network-wise language and then into rules readable by security enforcement systems. The translated policies are automatically loaded to the correspondent enforcement systems through a specifically designed module. Finally, we integrate an IDS with the policy translation system, in order to obtain an evaluation of the risk level, improving the policies generated and making them adequate to the organization’s requirements.

Our solution integrates different main systems which have been designed to focus on each one of the main functionalities of the solution: The high-level policy definition, the translation of policies to a low-level language, and finally, the integration of an intrusion detection system to improve the policy configuration, generating an automatic intrusion response system. In the following sections, we explain in detail each one of these systems and their functionalities.

### 3.1. High-Level Policy Definition

**Figure 2:**
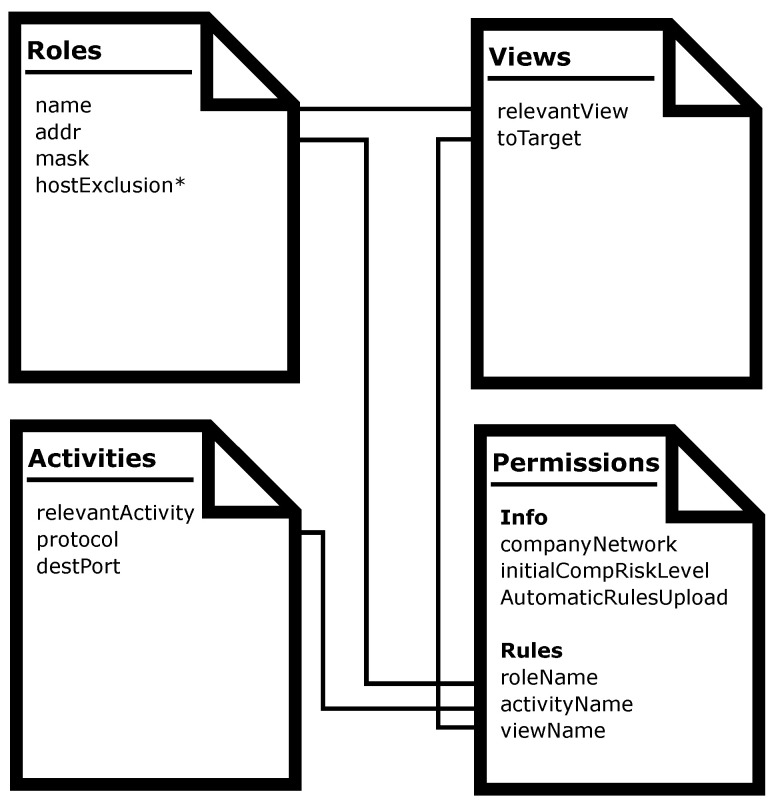
High level language eXtensible Markup Language (XML)-based documents, their main fields and their relationships.

As we explained in Section 1, we have defined a high-level security policy language based in an RBAC model, which is one of the most suitable for organizations’ networks, due to the role-based access scheme usually used in that kind of environments. The language, as some of the available policy definition alternatives, is based on XML. We have defined a series of files, fields, and labels to define roles, activities, views, and permissions. Figure 2 shows each document and the relationships between them. In the next sections, we will explain each one in detail.

#### 3.1.1. Roles Document

A Roles XML document must be defined. In this file, each role in the security scheme is stored. Each role must be composed at least by a name, a network address, and a mask.

The name field must follow a specific structure that will depend on the type of role defined. For instance, for firewalls it must start with “FW_”, then the name chosen for the role (an alphanumeric string), and finally a network interface name. There will be a role for each interface connected to the network, so any device with more than one interface will require the registration of a role for each one with the same role name and different interface name. There will be specific roles for the Internet access interface in the network and also for users. At least one administrator should be defined in each configuration. Network address and masks will correspond to the IP and mask of each interface.

In the Roles document, it is possible to mark some roles as excluded by marking them with a specific optional “hostExclusion” field. This is usually useful for excluding certain IP addresses and masks from the configuration.

#### 3.1.2. Activities Document

We define activities as the possible actions related to the organization business that makes use of the network and require security. Therefore, in the Activities document, there will be one entry for each activity to consider in security policies. These entries must be composed by three fields: The “relevantActivity” field, where the activity name is registered, the “protocol” field, that indicates the protocol used in that specific activity (UDP (User Datagram Protocol), TCP (Transmission Control Protocol), or ICMP (Internet Control Message Protocol)), and “destPort”, which indicates the destination port used if the chosen protocol is UDP or TCP.

#### 3.1.3. Views Document

This document determines which of the roles defined in the Roles document are going to be granted access in this configuration. It is composed of two fields. The first one, “relevantView” is used to define the view name and the second one, “toTarget”, links the role name used in the view.

#### 3.1.4. Permissions Document

The Permissions document is the file where the actual policies are defined. It is composed of two main sections: “Info” and “Rules”. The “Info” section is also composed of various labels which determine the information about the permissions. For instance, the organization’s network IP address and mask and the initial risk level assessed initially for the organization (a number between 0 and 10, being 10 the highest possible risk). We also included a label to indicate whether the rules associated with these permissions should be automatically uploaded and deployed to the network devices or not.

The “Rules” section, on the other hand, defines each permission in the system. As with the organization initial risk, each rule is assigned a number between 0 and 10 to determine the risk associated with it. Each rule links roles, activities, and views, as defined in the correspondent documents, generating a policy which can be later translated into the low-level abstract language.

### 3.2. Policy Translation

The translation process requires moving the policies from the high-level XML-based language to a more abstract language, closer to the devices in charge of enforcing the policies. We have designed the system to be technology agnostic. This means that, in a first phase, the goal low-level language will not be directly the one used by firewalls and other devices, but an abstract language that considers the network topology and the organization’s requirements to define the security policies. This abstract layer can be then translated again into the specific language understood by the devices in a second phase.

#### 3.2.1. Phase 1: High-Level Language to Abstract Language

For the first phase of the translation, the XML documents generated in the high-level language are examined, and, following a series of steps, the policies are transformed into the intermediate abstract technology-agnostic language which could later be applied to different devices through a second translation process. The steps followed during translation are:

1. There is an initial discovery and mapping process to determine which roles are relevant to which devices. This means that only roles reachable within one hop (that is, they share a subnet) from a firewall device in the network would be considered relevant. This discovery process is performed by checking IP addresses and masks of each role and comparing them to the IPs configured for each interface of a device. This process generates a new XML file called “FirewallRelevantRoles”, An example of the contents of this file would be:

<Firewall name="FW_2">
    <RelevantRoles>
        <role>DMZ</role>
        <role>FW2_internal</role>
        <role>FW2_external</role>
        <role>Web_server</role>
        <role>Admin</role>
    </RelevantRoles>
</Firewall>
  As the example shows, the firewall own interfaces are stored with the rest of the roles in the network. This information will be later used to determine which firewalls will be affected by a certain high-level policy.
2. For each set of permissions, the related roles are read and their information (IP and mask) is stored temporally. Then the “activityName” of the permission allows the method to find the corresponding activity in the “Activities” file, where the protocol and ports are specified.
3. Then, there is a translation process for permissions corresponding to the initial risk assessment of the organization. For each permission, the translator looks up for the role which wants access to the resource (“subjectRole”), the role of the resource being accessed (“viewRole”) and the type of access.
4. Once the rule has been processed, the firewall devices located between the “subjectRole” and the “viewRole” must be found. These devices are the ones that should incorporate this access rule in their configurations. This process is carried out by querying the routing elements in the network, considering that all routing devices act also as firewalls, and then using the initial discovery process to find out the firewalls closest to the beginning and the ending of the communication between roles. This task starts by looking in the “FirewallRelevantRoles” file the firewalls related to the “subjectRole”. The process will count on two firewall lists, one related to the “subjectRole” and other related to the “viewRole”, and, assuming the path is bidirectional, it starts querying the firewalls in the shortest list to reconstruct the path.
5. A query looking for the next hop is issued to each firewall device that can be a candidate to be the first in the communications path, in order to analyze the response. This way, it is possible to create a list of all the devices affected by the policy being translated. The querying of each network device is performed through the SSH protocol and requesting the routing configuration of each one regarding the destination subnet (that is, the “viewRole” or “subjectRole” network, depending on whether the process is using one list or the other). The firewalls will respond with the next-hop address in each case, or with the corresponding network interface if the firewall is located at the end of the path and no more hops are needed.
6. Once there is a list of all the network devices involved in the policy, it is possible to translate it, adding specific rules for each one of the devices. The translated rule will be composed of the source and destination addresses, the type of traffic and the ports used. Also, it will be related to the firewalls in use in that specific traffic flow.

This way, it is possible to generate a more detailed policy using an abstract language, also based in XML which holds enough information for it to be translated into specific rules for specific devices. The “AbstractionRules” XML file will contain the abstract rules translated from the high-level language. An example of this file would be:


<info>
    <subjectRole>Web_Server</subjectRole>
    <activity>Streaming</actitvity>
    <view>To_Internet</view>
    <companyNetworok>10.1.0.0/21</companyNetwork>
    <automaticRulesUpload>yes</automaticRUlesUpload>
    <adminIP>10.1.3.12/32</adminIP>
</info>


This file contains the above information about the specific subject role. Also, for each firewall were the rules must be installed will contain information such as:


<firewall IP="10.1.2.1/32" name="FW_1">
    <sourceIP>10.1.1.0</sourceIP>
    <sourceMask>21</sourceMask>
    <destinationIP>Internet</destinationIP>
    <destinationMask>0</destinationMask>
    <protocol>udp</protocol>
    <destinationPort>8000</destinationPort>
</firewall>
          

This first translation adds an abstract layer between the users and the network, enabling an easier configuration, without explicitly considering the network topology and configuration in the high-level policy definition.

#### 3.2.2. Phase 2: Abstract Language to Device Language

The second phase takes as input the abstract policy configuration obtained in the first phase and translate the rules into actual firewall rules. This translation process depends heavily on the specific control mechanisms and technologies installed in the network. In this paper, we have selected Iptables as the firewall tool, although any it could be substituted by any other similar technology solution, modifying only this second translation phase.

For each rule defined in the abstract language, this process creates an Iptables chain that can be later installed in the corresponding firewall and enforce the original policies configured by users.

Specifically, our solution takes each rule specified in the “AbstractionRules” XML file and generates an Iptables chain that matches it for each firewall. The chain name is composed of the “subjectRole”, the activity and the view corresponding with the rule. Then, reading the addresses, protocols, and ports from the XML file, it generates a series of Iptables rules. For instance, a set of rules for a given permission would be:


iptables -N FW_1-SSH-To_Admin
iptables -A FORWARD -s 111.222.100.1/32 -p tcp --dport 22 -j FW_1-SSH-To_Admin
iptables -A FW_1-SSH-To_Admin -d 111.222.2.20/32 -j ACCEPT
          

Ultimately, this second phase generates a set of rules that can be automatically deployed and enforced in Iptables-based firewalls. The independence between the high-level language, the abstract layer language, and the actual rules allow both an easy way for policy definition and the possibility of exchange the firewall technology without modifying the permissions set. Only the translation process between the abstract language should be reconsidered and rebuilt.

### 3.3. IDS and AIRS Integration

Our policy definition and configuration system is able to use the alerts generated by an IDS to improve the security policies. This integration is carried out by receiving TCP messages when there are intrusion alerts in the detection system. Depending on the type of intrusion, the risk assessment of the organization is modified, which, as a consequence, determines the rules to be deployed in the network from those defined in the high-level language, as it was explained in the second step defined in Section 3.2.1.

Regarding the automatic intrusion response system (AIRS), it is achieved by including an automatic process for the deployment of rules to the devices. This process is performed automatically through specific SSH (Secure SHell) connections using public key-based access to avoid user interaction. This process is carried out automatically in real-time, each time the policies need to be updated.

Although one of the main goals of our proposal is to automate as much as possible the deployment and enforcement of security policies, it is not always possible or desirable to fully automate the process. Depending on the requirements of the specific policies, or on the possible negative impact of them on the organization, certain permissions can be configured to require a human analyst approval before their deployment.

A huge amount of alerts can be generated by the IDS, and it is up to the specific IDS system to manage them adequately in terms of scalability and performance. The automatic rule deployment process depends on the risk assessment task, which will determine the risk depending on the alerts, historical data, and preset threat signatures. Only if a certain level of risk is achieved, will new policies be deployed, avoiding a high number of changes in security policies in the network. This also allows us to prevent or mitigate the possibility of deploying policies based on false-positive alerts from the IDS. In any case, if an alert is detected as a false positive, a new risk assessment will be calculated and the policies can be removed from the network devices.

## 4. Validation Scenarios

The validation of the proposed system has been addressed by the deployment of the whole translation and rule system over two testing scenarios, which represent the most common companies’ network layouts. Both scenarios consisted of two virtualized systems produced by VNX (Virtual Networks over linuX) virtualization platform [39]. In addition, a graphical user interface (GUI) has also been developed over the high-level language which enables users to configure and generate high-level XML documents without manual editing. Both the user interface and the translation process have been developed in Java. Finally, a simple bash script has been made to start the scenarios and to configure the NAT allowing the virtualized machines to access the internet to make the scenarios as realistic as possible:


#!/bin/bash
sudo vnx -f $1.xml -v -t
sudo vnx_config_nat INTERNET enp0s3
      

The virtualized firewalls used in the validation scenarios are virtual Linux machines running Iptables. As IDS we used the open-source solution Suricata [40].

### 4.1. Scenario A: Proof of Concept Test

**Figure 3:**
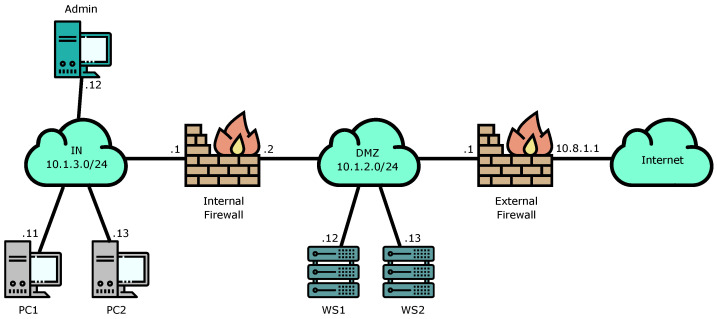
Topology of the first proof of concept scenario: An organization requires giving access from the internet to certain services while protecting the internal network. WS1 and WS2 are Web Servers located in the DMZ.

For the first validation scenario, we have designed a very common topology used in many organizations to offer internet-based services such as web pages while protecting the rest of their networks. It is based on the configuration of a DMZ (de-militarized zone) between internet access and the internal network. Firewalls are used to securely separate each network. A diagram showing the basic elements of this topology can be seen in Figure 3.

The initial risk assessed for the organization has been set to 0, the lowest in our scale, in this test. We have defined two specific permissions for that level:

- PC1 must be able to ping WS1 (Web Server 1) periodically to check if the server is running. This means that ICMP traffic must be allowed.
- Every device in the internal network (IN) must be able to access the Internet through ports 80, 8080 and 443 (that is, they should be able to use HTTP and HTTPS protocols). The Admin PC should not be given those permissions for security reasons.

From the high-level language this means that The following roles must be configured (IPs and masks are taken from the sample addresses shown in Figure 3):

- Name: WS1, IP: 10.1.2.12, Mask: 32
- Name: PC1, IP: 10.1.3.11, Mask: 32
- Name: Internet, IP: InternetIP, Mask: 0, ExcludedRoles: FW_ex_e
- Name: IN, IP: 10.1.3.0, Mask: 24, ExcludedRoles: FW_in_i, Admin

Two views will be defined for the devices used in this case, one for WS1 and one for the internet. Then, the activities defined are:

- Name: Web_HTTP, Protocol: TCP, Port: 80,8080,443
- Name: PING, Protocol: ICMP

And finally, the rules to implement the security requirements are:

- From: PC1, Allow: PING, To: WS1
- From: IN, Allow: Web_HTTP, To: Internet

The complete high-level configuration will also include the information about the initial risk assessment for the organization, the network subnet and the automatic uploading configuration.

This high-level policy configuration will then object to the first translation phase, which will generate the abstract language policies that consider the specific network topology and configuration. For instance, the already configured ICMP-related permission would result in this XML:

<Firewalls>
    <info>
        <subjectRole>PC1</subjectRole>
        <activity>PING</activity>
        <view>To_WS</view>
        <companyNetwork>10.1.0.0/22</companyNetwork>
        <automaticRulesUpload>yes</automaticRulesUpload>
        <adminIP>10.1.3.12/32</adminIp>
    </info>
    <Firewall IP="10.1.2.2/32" name="FW_in_i">
        <sourceIP>10.1.3.11</sourceIP>
        <sourceMask>32</sourceMask>
        <destinationIP>10.1.2.12</destinationIP>
        <destinationMask>32</destinationMask>
        <protocol>ICMP</protocol>
    </Firewall>
</Firewalls>
        

From this abstract XML, the second translation phase would generate the actual Iptables rules for each one of the permissions and for each one of the available firewalls. The rules for the first permission in the internal firewall once translated are:


iptables -N PC1-PING-To-WS1
iptables -A FORWARD -s 10.1.3.11/32 -p icmp -j PC1-PING-To_WS1
iptables -A PC1-PING-To_WS1 -d 10.1.2.12/32 -j ACCEPT
        

For the second permission they are:


iptables -N IN-Web_HTTP-To_Internet
iptables -A FORWARD -s 10.1.3.0/24 -p tcp --match multiport --dports 80,8080,443
    -j IN-Web_HTTP-To_Internet
iptables -A IN-Web_HTTP-To_Internet -s 10.1.3.1/32 -j RETURN
iptables -A IN-Web_HTTP-To_Internet -s 10.1.3.12/32 -j RETURN
iptables -A IN-Web_HTTP-To_Internet -d 10.8.1.1/32 -j RETURN
iptables -A IN-Web_HTTP-To_Internet -d 10.1.0.0/22 -j RETURN
iptables -A IN-Web_HTTP-To_Internet -j ACCEPT
        

In the external firewall, only rules regarding the second permission have to be installed, given that the first permission is only relevant to the internal network and the DMZ. Therefore, the translated rule will be:


iptables -N IN-Web_HTTP-To_Internet
iptables -A FORWARD -s 10.1.3.0/24 -p tcp  --match multiport --dports 80,8080,443
    -j IN-Web_HTTP-To_Internet
iptables -A IN-Web_HTTP-To_Internet -d 10.1.0.0/22 -j RETURN
iptables -A IN-Web_HTTP-To_Internet -j ACCEPT
        

All the translation process, from the high-level XML language to the Iptables rules, as well as the consequent installation and enforcement of the permissions, is fully automatic and does not require further interaction from the user managing the security policies. The system allows for the automatic selection of devices relevant to each specific policy, and generates security rules only for them, as this scenario shows.

### 4.2. Scenario B: A Large Organization Use Case

**Figure 4:**
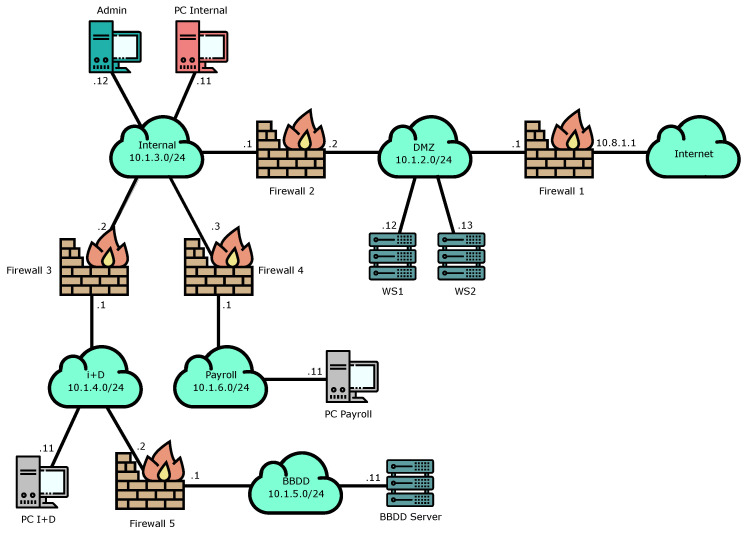
Topology of the second use case scenario: The organization needs to put in quarantine the internal network due to an alert of an employee (PC Internal) accessing a malicious site. DMZ is the DeMilitarized Zone network and BBDD is a database server.

We have selected the second validation test to be based on a more complex network topology and higher security requirements. In this sense, Scenario B represents a possible topology of a large company or organization with different departments and protected by five firewalls. The use case designed over this scenario is composed of four tests, one related to the roles management, second one oriented to the path discovery process, other related to quarantine management and final one oriented to manage an internal attack. A diagram of the topology used in this scenario is shown in Figure 4.

#### 4.2.1. Big Roles

The definition of roles in this access control method is not restricted to a given role “size”. This means that a role can identify from a single device or user to the whole organization. In this validation test, we define a role for the whole organization except the database network and the administrator machine. This way, it is possible, for instance, to receive UDP traffic in port 2343 by everyone in the network except for those two roles easily. The roles in this scenario would be:

- Name: Organization, IP: 10.1.0.0, Mask: 21, ExcludedRoles: BBDD, Admin
- Name: Internet, IP: Internet, Mask: 0, ExcludedRoles: FW_1_e
- Name: BBDD, IP: 10.1.5.0, Mask: 0
- Name: Admin, IP: 10.1.3.12, Mask: 32
- Name: FW_1, IP: 10.8.1.1, Mask: 32

The Activities file, in this case, will be composed of just one activity:

- Name: Stream, Protocol: UDP, Port: 2343

The view defined in this case is:

- Name: To_Internet, Role: Internet

And finally, the permission rule to be defined (with the associated risk level 0) is:

- From: Organization, Allow: STREAM, To: Internet

Once the rules are translated to the low-level firewall language, it is determined that, in this case, all the firewalls are related to the rule, and therefore, there will be Iptables rules for each one in the topology. For instance, the rules for firewall 5 would be:


iptables -N Organization-STREAM-To_internet
iptables -A FORWARD -s 10.1.0.0/21 -p udp --dport 2343
    -j Organization-STREAM-Internet
iptables -A Organization-STREAM-To_Internet -s 10.1.5.0/24 -j RETURN
iptables -A Organization-STREAM-To_Internet -s 10.1.3.12/32 -j RETURN
iptables -A Organization-STREAM-To_Internet -d 10.1.0.0/21 -j RETURN
iptables -A Organization-STREAM-To_Internet -j ACCEPT
          

Allowing all the traffic from the Organization network to the internet and refusing streams from the BBDD network or the administrator machine. Firewalls 2 to 4 would install similar rules, and for firewall 1 the rules would be:


iptables -N Organization-STREAM-To_Internet
iptables -A FORWARD -s 10.1.0.0/21 -p udp --dport 2343
    -j Organization-STREAM-To_Internet
iptables -A Organization-STREAM-To_Internet -s 10.1.5.0/24 -j RETURN
iptables -A Organization-STREAM-To_Internet -s 10.1.3.12/32 -j RETURN
iptables -A Organization-STREAM-To_Internet -d 10.8.1.1/32 -j RETURN
iptables -A Organization-STREAM-To_Internet -d 10.1.0.0/21 -j RETURN
iptables -A Organization-STREAM-To_Internet -j ACCEPT
          

The difference between this firewall and the others is that it includes a chain for blocking traffic to its external interface. This is automatically included because it was found in the excluded roles list for the internet role.

#### 4.2.2. Path Discovery

This validation test presents a scenario where there must be an access from the payroll department to the BBDD server. The goal of this test is to determine whether the rules are installed only to the relevant firewalls or not. As Figure 4 suggests, the firewalls used in this test should be FW_3, 4 and 5.

The roles in this case would be:

- Name: Payroll, IP: 10.1.6.0, Mask: 24, ExcludedRoles: FW_4_i
- Name: BBDD_server, IP: 10.1.5.11, Mask: 32

The Payroll role would represent the payroll department, excluding the internal interface of the correspondent firewall. The activity related to this permission would be:

- Name: CONSULT, Protocol: TCP, Port: 6001

To allow querying the database server using the 6001 port. The associated view is:

- Name: To_BBDD, Role: Payroll

And the rule:

- From: Payroll, Allow: CONSULT, To: BBDD

After the translation process, we can verify that only firewalls 4, 5 and 5 count with new Iptables rules. The rules for firewall 4 are:


iptables -N Payroll-CONSULT-To_BBDD_Server
iptables -A FORWARD -s 10.1.6.0/24 -p tcp --dport 6001
    -j Payroll-CONSULT-To_BBDD_Server
iptables -A Payroll-CONSULT-To_BBDD_Server -s 10.1.6.1/32 -j RETURN
iptables -A Payroll-CONSULT-To_BBDD_Server -d 10.1.5.11/32 -j ACCEPT
          

In firewalls 3 and 5 the rules inferred are:


iptables -N Payroll-CONSULT-To_BBDD_Server
iptables -A FORWARD -s 10.1.6.0/24 -p tcp --dport 6001
    -j Payroll-CONSULT-To_BBDD_Server
iptables -A Payroll-CONSULT-To_BBDD_Server -d 10.1.5.11/32 -j ACCEPT
          

The rules for firewall 4 are more strict because it is the first firewall in the path to the flow destination. This validation test shows how the system is able to determine the firewalls in the path and generate only the required rules for the specific devices in the network, avoiding the necessity for the user to determine by hand each rule for each firewall.

#### 4.2.3. Quarantine

In this scenario, we consider the information obtained by the integrated IDS. An alert issued by the detection system states that an employee from the “Internal” department is trying to access a site considered malicious, and therefore could have been infected. To prevent further damage in the organization’s network, the “Internal” department network must be put in quarantine. We consider that the potential attack is carried out from “PC Internal”, getting infected with a malware that tries to use FTP (File Transport Protocol) through the TCP port 20 to obtain information from the organization.

The test starts also with a level 0 risk assessed for the organization, but it is then analyzed again after receiving the alert, increasing it to a level 2. This means that the rules configured for the department isolation will be installed and enforced.

The configured high-level language rules for this scenario at risk level 0 (initial assessed risk in the scenario) are:

- From: Internal, Allow: Web_HTTP, To: Internet
- From: Internal, Allow: FTP, To: Internet
- From: Internet, Allow: Web_HTTP, To: DMZ
- From: FW_1_i, Allow: LOGSTASH, To: Admin

There are another two rules configured for risk level 2, which will be translated and uploaded to the correspondent firewalls after receiving the IDS alert, removing the risk 0 configuration and isolating the “Internal” department network:

- From: Internet, Allow: Web_HTTP, To: DMZ
- From: FW_1_i, Allow: LOGSTASH, To: Admin

The detection of the intrusion by the IDS, is carried out by the continuous monitoring of traffic through Firewall 1, where the system is deployed. The alert generated by the IDS is generated when the following rule is matched:


alert tcp 20.1.3.0/24 any -> 83.223.12.3 20 (msg:"Alert, FTP to suspicious IP,
level 2"; classtype: policy-violation; sid:2; rev:2;)
          

The rule has been automatically created by checking periodically malicious IP sites. The alert generated is saved to a log file and the “Logtash” module (a software tool for the management of logs) sends it to the administration machine (Admin). There, the alert is analyzed and the new risk level is determined according to it.

Once the risk is automatically increased, the new permissions are uploaded to the corresponding firewalls and the internal network becomes isolated. The whole process requires no human interaction (although the risk assessment can be human aided), making the translation and enforcement system behave as an AIRS.

#### 4.2.4. Internal Threat Management

In this last validation test, we define a scenario where there is an internal attack on the BBDD (Database) server. The goal is to determine how the system detects it and automatically modify the security policies to protect the organization’s data.

The attack will be run from a computer inside the Payroll department. A discontent employee might try to access the database server from his computer and delete all the organization’s information. The user might not know the database administration credentials, but try to access the server anyway.

Given that firewall 5 contains the IDS and is monitoring the traffic through it, it will detect the intrusion and will be considered as a potential risk to the organization. The risk assessment process determines that the current risk level should be raised to 7. The IDS rule might be:


alert tcp 10.1.6.0/24 any -> 10.1.5.11 15532 (msg:"Alert, reiterated failed BBDD log
in, level 7"; flags: S+; threshold: type both, track by_src, count 2, seconds 30;
classtype: policy-violation; sid:2; rev:2;)
          

That rule will be matched when someone from the payroll department tries more than one login attempt in less than 30 s to the BBD server. The initial risk level 0 already had some established permissions:

- From: Admin, Allow: MANIPULATE, To: BBDD_server
- From: Payroll Allow: MANIPULATE, To: BBDD_server
- From: Internal Allow: MANIPULATE, To: BBDD_server
- From: FW_5_e Allow: LOGSTASH, To: Admin

**Figure 5:**
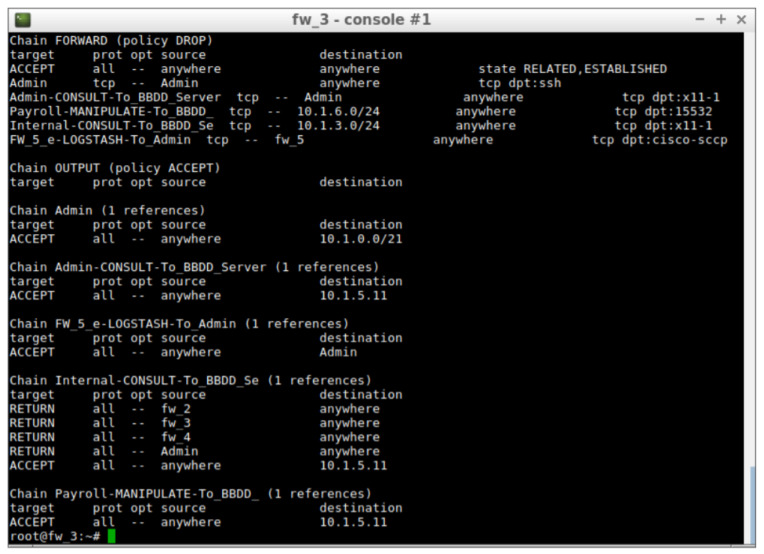
Rules installed in firewall 3 at risk level 0.

Which have led to the configuration of firewall rules in firewall 3. The rules are shown in Figure 5. These rules are directly related to the established permissions and there is an Iptables chain for each one.

After the internal attack is performed (it has been emulated by generating traffic from a PC in the payroll department), an alert is issued to the administrator computer and the risk is automatically increased to level 7.

The only established permission for risk level 7 is:

- From: FW_5_e Allow: LOGSTASH, To: Admin

**Figure 6:**
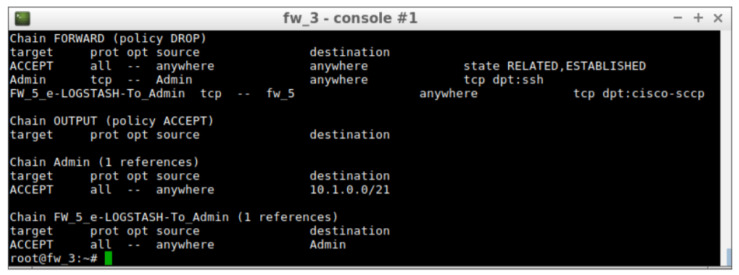
Rules installed in firewall 3 at risk level 7.

This allows automatic protection of the BBDD server by removing the permissions given in the risk level 0. After the modification of the security policies, the Iptables chains in Firewall 3 will be reduced to two, as shown in Figure 6.

The two rules left in the firewall allow the admin to configure firewalls and receive alerts from the IDS, but the access to the BBDD server has been completely restricted. The whole process has been performed automatically and will protect the servers until a new risk assessment is carried out.

## 5. Discussion and Conclusions

In this paper, we have proposed a solution to improve and facilitate the definition of the security policies in organizations’ networks. The main goal of this proposal is to offer an easy-to-use high-level definition language to users which can be then translated into specific rules using a completely automated process for its deployment and enforcement.

This XML-based high-level language has been designed to be technology-agnostic and not dependant on the specific network topologies where it is going to be applied, which contributes to the ease of policy definition from the user perspective. The two-phase translation allows the generation of an intermediate set of permissions in an abstract language which is dependant on the actual network topology without any user interaction, but still agnostic from the specific security tools used as firewalls in the network. This means that it would be possible to use different devices and tools without modifying the abstract permissions defined. Only the phase two of the translation process should be run again, updated to the new language understood by the devices deployed in the network.

In order to automate as much as possible the whole system, the definition of policies has been attached to a risk assessment process, which determines, in each moment, which policies should be deployed and enforced. The risk assessment is carried out using the information given by an integrated intrusion detection system that sends alerts when there are possible security breaches detected. The policy translation and enforcement system act, in consequence, depending on the current risk level, behaves as an automatic intrusion response system, and therefore integrates into a single system the translation and installation of policies, the intrusion detection, and the dynamic response to the threats found.

One of the main improvements of our approach, when compared with other response systems, is the integration of the policy definition language with the automatic response to intrusions, which makes the system even more autonomous and easy to configure. Also, automating the process of rule generation, deployment and enforcement makes the whole security system less prone to mistakes or errors which could cause important damage to the organization.

The system has been developed and tested in virtual networks defined specifically to test the features of the proposal. The first test, a simple security configuration for a organization’s intranet protected by a DMZ, has validated the approach, by showing how defining a few lines of text (which are even easier to configure through a specifically developed GUI) makes it possible to successfully protect an internal network from unwanted accesses from the internet.

Our second test scenario, composed of four different validation tests, has shown how the roles in our scheme might be defined to group as many elements in the network as needed for the simplification of permissions, how the translation process determines the relevant devices for each permission and only generate rules from them, the integration of the policy translation system with an IDS to generate a complete AIRS, and an example for the detection and reaction to internal threats. The integrated system improves the security of the scenario without needing explicit user interaction. The policies configured as a response to certain security issues (for instance, the isolation of an internal network as shown in the third test, can be already configured and waiting for the correspondent alert from the IDS to be automatically deployed and enforced if the risk level is considered high enough. The translation and deployment process, despite its flexibility, it is simple enough to be accomplished in a short time, avoiding time gaps between threat detection and the new policy deployment.

In this paper, we have focused our efforts specifically in the generation of access control policies, but the scalable and modular nature of the high-level language would allow the inclusion of further syntax to extend it to other security dimensions such as confidentiality, integrity or availability. This would require the translation of these high-level permissions into additional security services covering those dimensions, but the process would remain as presented in this paper. Also, the integration with the IDS and the risk assessment process would remain as shown in this work and would be used for the automatic deployment of rules in the specific devices, depending on their relationship with the permissions goals.

As future work lines for this research, we intend to deploy the system in a real test network, with the goal of experimenting with different configurations and intrusions, to be able to test how the automatic response system behaves in different scenarios. Additionally, the high-level policy definition language and its translation process will be studied further to include more flexibility in the available security configurations. For instance, the possibility of include user-centered security policies based on user preferences and regardless of the actual organization’s network map, while considering the organization requirements in terms of security.

From the development point of view, we plan to implement more phase 2 translators from the abstract language to different firewall technologies beyond Iptables, allowing the system to be deployed in a wider range of networks with heterogeneous security devices and covering further security dimensions. We also plan to perform tests to quantify the specific time overhead produced by the translation and automatic deployment in realistic scenarios. Finally, it is planned to address the formal verification of the proposed translation model to extend the evaluation of the proposed solution and thus analyze its accuracy and robustness.

## Abbreviations

The following abbreviations are used in this manuscript:

AAIRS	Adaptive Agent-Based Intrusion Response System
ABAC	Attribute-Based Access Control
AIRS	Automatic Intrusion Response System
ASL	Authorization Specification Language
CapBAC	Capabilities-Based Access Control
CPBAC	Conditional Puropose-Based Access Control
DDoS	Distributed Denial of Service
DMZ	DeMilitarized Zone
DRM	Digital Rights Management
EPAL	Enterprise Privacy Authorization Language
EPL	Event Processing Language
FTP	File Transfer Protocol
GUI	Graphical User Interface
HIDS	Host-based Intrusion Detection System
ICMP	Internet Control Message Protocol
IDS	Intrusion Detection System
IF	Intermediate Format
OrBAC	Organization-Based Access Control
OSL	Obligation Specification Language
OSSEC	Open Source HIDS SECurity
OSSIM	Open Source Security Information Management
RBAC	Role-Based Access Control
SDN	Software Defined Networking
SIEM	Security Information and Event Management
SSH	Secure SHell
TCP	Transport Control Protocol
UDP	User Datagram Protocol
VALID	Virtualization Assurance Language for Isolation and Deployment
VNX	Virtual Networks over linuX
XACML	eXtensible Access Control Markup Language
XML	eXtensible Markup Language
